# Impact of exercise training on cardiotoxicity and cardiac health outcomes in women with breast cancer anthracycline chemotherapy: a study protocol for a randomized controlled trial

**DOI:** 10.1186/s13063-019-3499-9

**Published:** 2019-07-15

**Authors:** Pedro Antunes, Dulce Esteves, Célia Nunes, Francisco Sampaio, António Ascensão, Eduardo Vilela, Madalena Teixeira, Anabela Leal Amarelo, Ana Joaquim

**Affiliations:** 1 Research Center in Sport Sciences, Health and Human Development (CIDESD), Sport Sciences Department, Universidade da Beira Interior, Convento de Santo António, Rua Mateus Fernandes Lote 5 n° 37 1° C, 6201-001 Covilhã, Portugal; 2 Associação de Cuidados de Suporte em Oncologia, Rua Quintã, 4520-531 Sanfins, Portugal; 3 Mathematics Department, Universidade da Beira Interior, Convento de Santo António, 6201-001 Covilhã, Portugal; 4 Oncology Department, Centro Hospitalar Vila Nova de Gaia/ Espinho, Rua Conceição Fernandes, 4434-502 Vila Nova Gaia, Portugal; 5 Cardiology Department, Centro Hospitalar Vila Nova de Gaia/ Espinho, Rua Conceição Fernandes, 4434-502 Vila Nova Gaia, Portugal; 6 LaMetEx - Laboratory of Metabolism and Exercise, Faculty of Sport, University of Porto, Porto, Portugal; 7 CIAFEL - Research Centre in Physical Activity, Health and Leisure Department of Sports Biology, Faculty of Sports, University of Porto, Porto, Portugal

**Keywords:** Breast cancer, Cardiotoxicity, Cardiac healthcare, Supervised exercise, Supportive cancer care

## Abstract

**Background:**

Anthracyclines are chemotherapeutic agents frequently used in breast cancer (BC) treatment. Although it improves disease-free and overall survival, the use of anthracyclines is associated with a cumulative risk of cardiac toxicity. Preventive strategies to optimize cardiac health are needed and exercise is proposed as a potential non-pharmacological approach for counteracting anthracycline-related cardiotoxicity (ARC). Most of the data on the effects of exercise to reduce ACT are from animal studies, with only a few studies in a limited number of patients indicating beneficial effects. To better understand the effectiveness of exercise in the mitigation of ARC, clinical, real-world trials claim require a larger sample size and more accurate and valuable clinical biomarkers. In this study, we intend to include a large sample and investigate cardiac function through serial measures of biomarkers and imaging techniques.

**Methods:**

This protocol describes a two-arm, prospective, randomized controlled trial that will explore the cardioprotective effect of a structured exercise program in women with BC undergoing anthracycline-containing chemotherapy (ACT). Ninety adult women with early BC and recommended to receive ACT will be randomly assigned (1:1) to an intervention group or a control group. Patients allocated to the intervention group will perform a supervised exercise program three times per week, consisting of a combination of aerobic and resistance training with progressive intensity and volume, during the time period they receive ACT. The control group will receive standard BC care. Primary outcomes related to cardiac (dys)function will be circulating N-terminal pro-brain natriuretic peptide (NT-proBNP) levels, resting left ventricular (LV) longitudinal strain, and resting LV ejection fraction. Secondary outcomes will include the assessment of resting blood pressure, resting heart rate (HR), resting HR variability (HRV), recovery HR, physical function outcomes, self-reported physical activity level, health-related quality of life, and fatigue. Data will be obtained at baseline (t_0_), after the end of anthracycline-treatment (t_2_), and 3 months after t2 (t_3_). Additionally, NT-proBNP will be measured 1–24 h prior to each anthracycline-treatment cycle (t1).

**Discussion:**

The implementation of the present study design, using novel clinical biomarkers, will determine the effect of structured exercise interventions at mitigating ARC, with the overall aim of finding means to further improve BC care.

**Trial registration:**

ISRCTN, ISRCTN32617901. Registered on 24 October 2018. Last updated on 11 January 2019.

**Electronic supplementary material:**

The online version of this article (10.1186/s13063-019-3499-9) contains supplementary material, which is available to authorized users.

## Background

Over the last three decades the epidemiology of breast cancer (BC) has been marked by the clear increase in survival rates [1]. The accessibility of screening and the discovery of new therapeutic options are among some relevant factors related to the improved management of cancer. However, despite their undeniable clinical importance, anti-cancer treatments are also associated with frequent induction of side effects. Among those, cardiotoxicity emerges as a major challenge, limiting treatment options [2] and contributing to morbidity and mortality in this patient population [3–5].

In the BC setting, cardiotoxicity is typically associated with exposure to traditional cytotoxic therapies and particularly with the use of anthracyclines [6]. Anthracyclines are important and effective chemotherapeutic agents, frequently administered in curative and palliative regimens for BC, although their clinical use is limited by cardiac dysfunction, usually seen as cardiotoxicity [7]. The American Society of Echocardiography and European Association of Cardiovascular Imaging define cardiotoxicity as a decrease in left ventricular ejection fraction (LVEF) of > 10% to a value of < 53% [8]. However, it has been proposed that anthracycline-related cardiotoxicity (ARC) is a continuous [9] and dose-dependent phenomenon [10] that starts with acute myocardial damage [11], which can be detected by elevation of circulating cardiac biomarkers [12] and by impairment of left ventricular (LV) longitudinal strain [13], in turn preceding the commonly reported progressive decline in LVEF [9]. ARC may ultimately lead to overt heart failure, particularly when underestimated, not prevented or insufficiently prevented, or untreated, [9].

Unfortunately, and in addition to the manifestations associated with anthracycline treatment, BC survivors often exhibit a phenotype characterized by the presence of risk factors for cardiovascular disease (CVD), including advanced age, obesity, prior CVD, poor cardiorespiratory fitness, and inappropriate lifestyle (smoking, alcoholism, and sedentary lifestyle) [14, 15]. It is therefore not surprising that when compared to healthy individuals of a similar age, these patients have higher prevalence of CVD and greater mortality [4]. Considering the high risk of cardiac dysfunction, the study of preventive strategies is an emerging and unmet need, as these strategies may mitigate cardiac damage associated with the use of cardiotoxic agents and could lead to improvements in overall cardiovascular health.

In adults with CVD without cancer, exercise training is recognized as an important approach in cardiac rehabilitation [16]. Particularly, two meta-analyses have shown favorable effects of exercise on attenuation of N-terminal pro-brain natriuretic peptide (NT-proBNP) [17] and mitigation of LV remodeling [18] in patients with heart failure. Also, in the oncological setting, it has become clear that exercise is a safe and effective supportive therapy in the management of several treatment-related side effects and in improving overall physical fitness [19, 20]. Furthermore, in a recent position paper the European Society of Cardiology task force for cancer treatments and cardiovascular toxicity suggests the possible utility of aerobic exercise as a promising strategy to attenuate ARC [21]. However, this position remains to be complementarily analyzed as the evidence so far is mainly from studies in animal models [22, 23], with only a few studies in a limited number of patients to evaluate the cardioprotective role of exercise in cardiac function among women with breast cancer receiving cardiotoxic treatments [24–26]. Further clinical studies involving a large sample size and accurate clinical biomarkers are thus warranted to better understand the effectiveness of exercise in the mitigation of ARC.

### Hypothesis

The primary aim of this study is to ascertain whether a structured exercise program mitigates ARC, measured by change in the levels of circulating biomarkers (NT-proBNP) and cardiac (dys)function endpoints (LV global longitudinal strain and LVEF). The secondary aim is to evaluate the effectiveness of the intervention in the regulation of some cardiac health parameters: resting blood pressure, resting heart rate (HR), resting HR variability (HRV) and recovery HR. As exploratory objectives, we will assess physical function (cardiorespiratory capacity, upper limb strength, and lower limb functionality), self-reported physical activity level, health-related quality of life (HR-QOL), and fatigue. We hypothesize that exercise may limit the degradation of cardiac function and structure and benefit cardiac outcomes. We also believe that patients in the intervention group will improve overall physical fitness, HR-QOL, decrease their perception of fatigue, and increase physical activity levels.

## Methods/design

### Study design

**Fig. 1:**
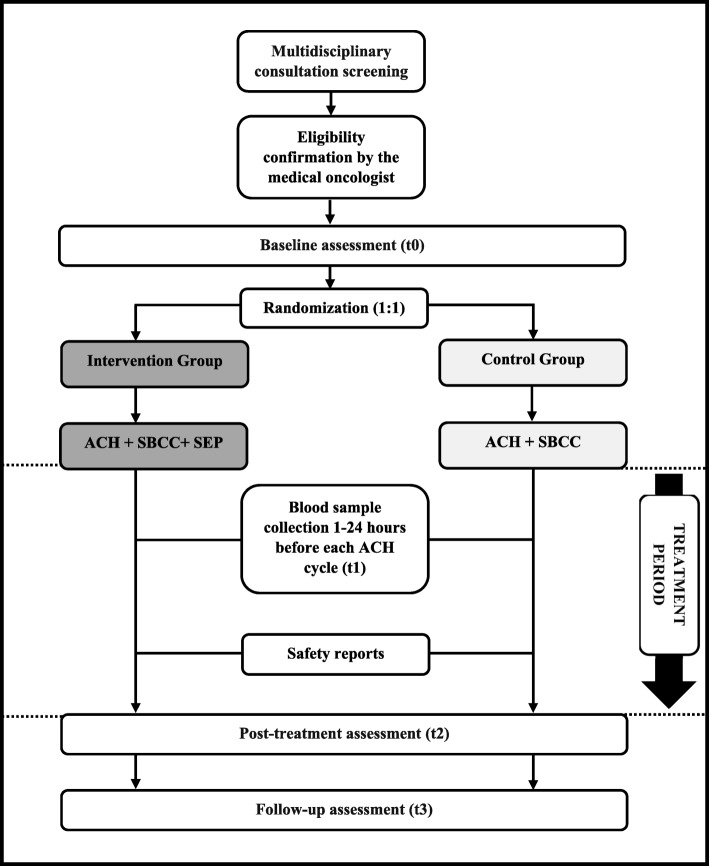
Study flow. ACH, anthracycline-containing chemotherapy; SBCC, standard breast cancer care; SEP, structured exercise program

This is a study protocol for a two-arm, prospective, randomized controlled trial that will explore the cardioprotective effect of a structured exercise program, compared to standard care, in adult women undergoing anthracycline-containing chemotherapy (AC-CT) for early BC. The study design and protocol adhere to the Standard Protocol Items: Recommendations for Interventional Trials (SPIRIT) guidelines (Additional file 1). The study design is outlined in Fig. 1.

### Ethical approval

This study will be conducted in compliance with the Declaration of Helsinki Ethical Principles (1975) and it received approval by the Ethics Committee of the Centro Hospitalar de Vila Nova de Gaia/Espinho (CHVNG/E; Vila Nova de Gaia, Portugal) (reference number: 145/2018–1). The study is registered in the International Standard Randomised Controlled Trial Number (ISRCTN32617901). Any protocol amendments will be submitted to the CHVNG/E for ethical approval and updated on the ISRCTN.

### Participant recruitment

We intend to recruit 90 adult women with early invasive BC, scheduled to receive AC-CT and followed up in the Medical Oncology Department of the CHVNG/E. Participants will be recruited based on the eligibility criteria presented in Table 1. Recruitment will take place in two distinct phases. In the first instance, potential participants will be identified in the multidisciplinary consultation involving medical oncologists, surgeons, and radio-oncologists. After this preliminary phase, the eligibility of each patient will be confirmed by the oncologist during a medical consultation. The oncologist will present the study to the patients who are considered eligible, explaining the study, offering participation and requesting written informed consent. Written informed consent will be obtained from all patients and they will be informed that they are under no obligation to participate and they may withdraw their consent at any time. Withdrawal from the study or non-participation will have no consequences for medical follow up and care. Where possible, the reasons for withdrawal from the study will be recorded. All the participants will be followed from the acceptance period (t0) and 3 months after the end of the AC-CT (t3). If recruitment is not achieving the target sample size, we will extend recruitment to additional hospitals.

**Table 1:** Eligibility criteria

Inclusion criteria	Exclusion criteria
Female gender	Contraindications to maximal exercise testing Decompensated diabetes mellitus
Aged 18 years or older	Severe anemia (hemoglobin < 8 g/dL) uncorrectable with transfusion and/or iron and/or vitamin deficiency replacement Pregnancy
Histological diagnosis of stage IA–IIIC breast carcinoma	Known significant heart disease (myocardial infarction, congestive heart failure, cardiomyopathy)
Scheduled to receive AC-CT	Usual medication containing beta-blockers
Follow up at the Medical Oncology clinic at the CHVNG/E	
Consent of the assistant oncologist for the practice of exercise	
Able to provide informed consent	
Acceptance of randomization to intervention group or control group	

*AC-CT* anthracycline-containing chemotherapy, *CHVNG/E* Centro Hospitalar de Vila Nova de Gaia/Espinho

### Randomization

After confirmation of eligibility and baseline assessments (t0), patients will be randomized using Internet software (www.sealedenvelope.com), in a 1:1 ratio to the supervised exercise group (intervention group) or the usual care group (control group), using a permutated block design with random block sizes (4, 6, 8) with stratification by two dichotomous variables, which are known risk factors for ARC:

- Age (< 50 years or older)
- Receiving anti human epidermal growth factor receptor 2 (anti-HER2) therapy (yes/no)

This process will be performed by an external individual who is blinded to the study and who will place the sequence in a numbered, opaque, sealed envelope. The allocation of participants will then be reported to an oncologist (AJ) who will subsequently inform the patients about the assignment group.

### Study arms

#### Intervention group

Patients allocated to the intervention group will perform a supervised exercise program specifically developed for patients with BC, based on the guidelines of the American College of Sports Medicine [27] and in a close cooperation between physical sports researchers (PA, DE, AA) and medical staff (oncologists, surgeons, radiologists, physiatrist, and physiotherapist) of the CHVNG/E. The exercise program comprises three sessions per week guided in small groups (< 5 patients) in an appropriately equipped room at the CHVNG/E, supervised by the main author (PA) and a physiotherapist. Each session will involve an initial warm up (5 min), followed by resistance and aerobic training (60 min), and ending with a cooldown phase (5 min). The program will be started 1–2 days after the first AC-CT session and will be conducted over the respective treatment of each patient. It should be noted that the proposed exercise intervention will never be intended to replace or interfere with the current standard BC care.

##### Aerobic training

It will include the combination of treadmill, stationary bike, and stepping. This phase will be monitored through HR (each participant will wear a HR monitor during exercise training sessions) and rate of perceived exertion (RPE) measured by a 0–10 point modified Borg scale (minimal effort = 0; maximum effort = 10) [29]. During the first 2 weeks, the participants will perform 20 min (divided equally among the three exercise modes) of light-intensity, aerobic training (at < 50% of measured HR reserve (based on maximum HR reached in the cardiorespiratory test), with a reported rating of 2–4 (“easy” to “somewhat easy”) on the modified Borg scale). After this period, 3 min will be added every 2 weeks until a volume of 30 min of aerobic training is reached. At this stage, participants will be encouraged to perform moderate-intensity to high-intensity training (at 65–80% of measured HR reserve, with a reported rating of 5–8 (“somewhat hard” to “hard”) on the modified Borg scale) until the end of the intervention.

Participants will be reminded weekly (by email and phone) of their exercise training schedule and the importance of adherence, to achieve the established objectives.

##### Resistance training

Resistance training will include upper body (shoulder press, chest press, lateral pulldown, biceps curls, and triceps extension) and lower body (squat, calf raise, leg press, leg extension, and leg curl) weight-training exercises. All the exercises will be performed at the maximum possible joint range of motion, using resistance machines and free weights. RPE will be measured using a 0–10-point OMNI-Resistance Exercise Scale (OMNI-RES, minimal effort = 0; maximum effort = 10) [28]. During the first week, participants will perform 2 sets and 10 repetitions of each exercise without additional resistance or with the lowest available (reported rating of 2–4 (“easy” to “somewhat easy”) on the OMNI-RES). After this phase, if no adverse events or symptoms were reported for a specific exercise, resistance will be added so that each participant could be able to perform 3 sets with 12 maximal repetitions (12-RM) of each exercise. When the participants can complete 3 sets and more than 12-RM at the set weight in three consecutive sessions, then the resistance will be increased by between 5% and 10%.

### Usual care group

Patients allocated to the usual care group (CG) will receive standard BC care. The usual CG will not receive any specific advice about physical activity and will not be asked to be inactive. In compensation for participation in this study, patients in this group will be offered the possibility to follow the same exercise program as in the intervention group, after the final assessments are completed.

### Study assessments

**Fig. 2:**
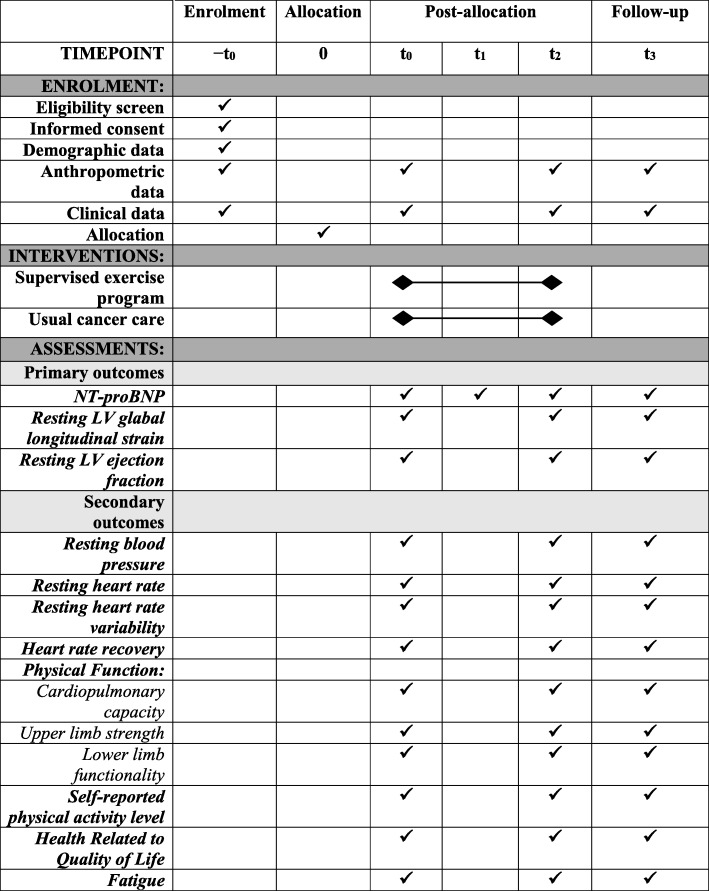
The schedule of enrolment, interventions, and assessments. -t_0_, enrolment process; t_0_, baseline; t_1_, during anthracycline-containing chemotherapy; t_2_. post anthracycline-containing chemotherapy (which coincides with the end of the intervention); t_3_, 3 months after t_2_; NT-proBNP, N-Terminal pro-brain natriuretic peptide; LV, left ventricular

The schedule of study outcome assessments is outlined in Fig. 2. Study assessments will be scheduled upfront and participants will be regularly reminded (by email and phone) to ensure complete follow up. Primary, secondary, and exploratory outcomes will be measured in all participants at three different time points:

- t_0_ (baseline assessments): between 0 and 14 days prior to the first chemotherapy session
- t_2_ (post-treatment assessments): between 1 and 5 days after the end of AC-CT
- t_3_ (follow-up assessments): after 3 months of t_2_

In addition, for analysis of circulating NT-proBNP, blood samples will be collected between 1 and 24 h before each AC-CT cycle (t_1_: during treatment assessments). Patients will be instructed and reminded to avoid drinking beverages containing alcoholic or caffeine, and to abstain from smoking for 12 h prior to, and avoid vigorous physical activities for 24 h prior to all examinations.

### Study outcomes

#### Primary outcomes

The primary outcomes are:

- Circulating NT-proBNP levels
- Resting LV global longitudinal strain
- Resting LV ejection fraction

#### Secondary outcomes

The secondary outcomes are:

- Cardiac health outcomes:
  - Resting blood pressure
  - Resting HR
  - Resting HRV
  - Recovery HR
- Physical function
  - Cardiorespiratory fitness
  - Upper limb strength
  - Lower limb functionality
- Self-reported physical activity level
- Health-related quality of life
- Fatigue

### Assessment of the primary outcomes

#### Resting LV ejection fraction and resting LV longitudinal strain

Resting LV ejection fraction will be calculated using the biplane method of disks (modified Simpson’s rule) from the apical four-chamber and two-chamber view [30]. For resting LV global longitudinal strain assessment, two-dimensional grayscale images will be acquired in the apical four-chamber, two-chamber, and three-chamber views, with a frame rate of 60–100 frames per s (fps). Three cardiac cycles will be digitally stored and Velocity Vector Imaging (VVI) software (Siemens Medical Solutions United States of America Inc) will be used in the analysis. Echocardiographic acquisitions will be performed by a single experienced cardiologist blinded to the patient assignment group.

#### Circulating NT-proBNP levels

Non-fasting venous blood samples will be drawn by a nurse oncologist. The assessment of NT-proBNP levels will be conducted in the local clinical analysis laboratories at the CHVNG/E, which are certified by the UK National External Quality Assessment Service. These professional staff will be blinded to the patient assignment group.

### Assessment of the secondary outcomes

#### Resting blood pressure and resting HR

Resting blood pressure (systolic and diastolic blood pressure) and resting HR will be measured using a standard automated device (Philips SureSignsVM6 (Philips Medical System, Andover, USA)). Two measurements will be carried out. The first measurement will be preceded by a 5-min resting period and a second reading will be taken after 3 min. If necessary, additional records shall be obtained until two consecutive stable measurements (differences < 5 mmHg for blood pressure and < 7 bpm for HR) are obtained. The average of the two stable measurements will be used in the analysis. This procedure will be carried out by a study investigator (ALA) not blinded to the patient assignment group.

#### Resting HRV

HRV is a non-invasive method used to analyze cardiac autonomic function through the measurement of successive heartbeat variations (R-R). Resting HRV will be analyzed using a HR monitor Polar V800 (Polar Electro Oy, Kempele, Finland) with a Polar H7 chest strap. During the R-R recording, patients will be seated in a comfortable position. They shall be required to breathe spontaneously, to avoid any movements, and to maintain neutral thoughts during the data acquisition. The first 5 min will be excluded (stabilization period) and the remaining 5 min will be used to calculate the time-domain (standard deviation of successive normal R-R (SDNN), and root mean square of successive normal R-R (RMSSD)) and frequency-domain indices (low-frequency spectral component (LF), and high-frequency spectral component (HF)). In all cases, the R-R recordings will be exported to the Kubios v2 HRV software (Biosignal Analysis and Medical Imaging Group at the Department of Applied Physics, University of Kuopio, Kuopio, Finland). Occasional artifact noise shall be automatically replaced with the interpolated adjacent RR interval values (filter power < low). This procedure will be carried out by the first author (PA) who will not be blinded to the patient assignment group.

#### Recovery HR

Recovery HR will be determined as the absolute difference between the HR at peak effort during the cardiorespiratory exercise test (CRET) and the HR at 60 s, and 120 s post-exercise. HR values will be derived from a continuous record obtained via CRET (Mortara X-Scribe, Mortara, USA). This procedure will be carried out by study investigators (EV, MT), blinded to the patient assignment group.

#### Cardiorespiratory fitness

Cardiorespiratory fitness will be evaluated by means of a symptom-limited CRET on a treadmill (Mortara X-Scribe, Mortara, USA), using a modified version of the Bruce protocol [31]. Expired gases will be continuously collected throughout exercise and analyzed for ventilatory volume (VE) and for oxygen (O2) and carbon dioxide (CO2) content, using dedicated analyzers. Standard spirometry (forced expiratory volume in 1 s (FEV1)) and forced vital capacity (FVC) will also be undertaken before the test. Equipment calibration and measurements will be in accordance with the recommendations of the American Thoracic Society and American College of Chest Physicians [32]. The following parameters will be calculated and considered for analysis: peak oxygen consumption (peak V̇o_2_, measured in milliliters per kilogram per minute), peak respiratory exchange ratio (RER), defined by the ratio of CO2 production to O2 consumption at peak effort, oxygen consumption at the anaerobic threshold (AT), defined as the point at which CO2 production increases disproportionately in relation to O2 consumption, obtained from a graph plotting O2 consumption against CO2 production, and total exercise duration (measured in seconds). The maximum HR achieved will also be recorded. The CRET will be conducted on a separate day to the remaining outcomes assessments out by study investigators (EV, MT) blinded to the patient assignment group. Patients will be in a fasted state and will not be asked to discontinue current medication before the test.

#### Upper limb strength

Upper limb strength will be evaluated by maximal voluntary grip strength (measured in kilograms), using a digital handgrip dynamometer (Saehan Corporation, Masan, South Korea - model SH5003). Each subject will perform six trials, three in each arm, with an alternating bilateral sequence. The results will based on the average of the three trials, respectively. This procedure will be carried out by the first author (PA), not blinded to the patient assignment group.

#### Lower limb functionality

Lower limb functionality will be evaluated by the sit-to-stand test using a straight-backed chair (40 cm high). Each subject will be required to keep plantar support while flat on the floor with arms crossed at the chest, and sit and stand as many times as possible for 30 s. The score for the test will be determined by the number of repetitions achieved during this procedure. The procedure will be carried out by the first author (PA), not blinded to the patient assignment group.

### Self-reported physical activity level

The International Physical Activity Questionnaire-Short (IPAQ-SF) will be used to calculate the metabolic equivalent (MET) minutes per week spent in walking and in moderate and vigorous activities. Sedentary behavior will be determined based on time spent sitting per day (minutes). Based on their scores, participants will be categorized as having a low, moderate, or high physical-activity level. The Portuguese language version of the IPAQ-SF will be used [33]. Scoring will be analyzed by the first author (PA), not blinded to the patient assignment group.

#### Health-related quality of life and fatigue

The European Organization for Research and Treatment in Cancer (EORTC) Quality of Life C-30 (QOL-C30) is a self-administered, validated questionnaire to assess HR-QOL in patients with cancer [34]. It is composed of nine multi-item scales: five functional scales (physical, role, cognitive, emotional, and social), three symptom scales (fatigue, pain, and nausea and vomiting), and a global health and quality-of-life scale. Additionally, there are five single items of symptoms commonly reported by patients with cancer (dyspnea, sleep disturbance, appetite loss, constipation, and diarrhea), and an item that evaluates the perceived financial impact of the disease. In this study, the third version of this questionnaire will be used, in the Portuguese language [35]. Analyses will include the five functional scales, fatigue scale, and the global health and quality-of-life scale. The scoring of the several scales will be carried out by the first author (PA), not blinded to the patient assignment group.

### Demographic, anthropometric, and clinical data

Demographic, anthropometric, and clinical data will be recorded during the enrolment process (**−**t_0_). Demographic data include age, sex, and education. Anthropometric data include weight, height, and body mass index. Clinical data include disease, treatment information, past medical history, and current medication. These data will be extracted from the patients’ electronic medical files by two study coordinators (ALA, AJ) not blinded to the patient assignment group.

### Safety

The safety of the intervention will be assessed by weekly tracking and monitoring the number of adverse events according to the National Cancer Institute Common Terminology Criteria for Adverse Events version 4.0 (https://ctep.cancer.gov/protocoldevelopment/electronic_applications/ctc.htm). A meeting between the study investigators will be held weekly to review and discuss the reported adverse events. All serious adverse events will be immediately reported to the CHVNG/E ethics commission and to all study members, and will be reported in the study results. Adverse events will be evaluated by the study investigators who will make the decision to stop the study early if there is a clinically relevant increased risk.

### Data management

Study data will be managed by two study investigators (PA, AJ) using a predesigned data collection form (Microsoft Office Excel version 2016 (Microsoft Corporation, Redmond, WA, USA) and Statistical Package for the Social Sciences files version 23.0 (IBM Corporation, Armonk, NY, USA)) with double data-entry. Data checks will be regularly performed to ensure data quality. Patients will be identified by codes to ensure their anonymity, and only the authors involved in the trial will have access to their full identification details. The total number of patients who meet the study eligibility criteria will be recorded, as will the number of patients who agree or not agree to participate in the study, the number of patients assigned to each study arm, the number of patients who participate in all sessions, the attendance of each patient in the intervention sessions, the number of patients who provide follow-up data, the number of patients included in the final analysis, and the number of withdrawals.

### Sample size calculation

The sample size was determined by a power calculation based on resting LVEF outcome, using a non-commercial statistical power analysis program (G*Power Version 3.1.9.2). Based on an effect size of 0.6 for resting LVEF presented in a previous study [26], to ensure statistical power of 80% and a significance level of 0.05, using the *t* test for two independent groups, the recruitment of 72 participants is required. Predicting a 20% dropout rate [19], we estimate that a total of 90 patients will be needed (45 in each arm). There are no planned early stopping rules. Adverse events will be evaluated by the study investigators, who will make the decision to stop the study early if there is a clinically relevant increased risk.

### Statistical analyses

Statistical data analysis will be performed using the Statistical Package for the Social Sciences. The statistical significance will be set at a *p* value <0.05. Intention-to-treat and per-protocol approaches will be used both for all analyses. Data analysis will start with standard descriptive methods to describe the data (means and standard deviations will be calculated for continuous variables and absolute and relative frequencies for categorical variables).

Continuous variables will be compared between the two study groups using one-way analysis of covariance (ANCOVA), adjusted for the effect of the baseline values (covariate). A linear two-way mixed ANCOVA model with repeated measures (t1, t2 and t3) will be performed to test the difference over time between the two study groups and interaction (Group × Time), on primary and secondary outcomes, with the same covariate as the one-way ANCOVA. Bonferroni’s post-hoc procedure will be performed to locate the pairwise differences. Normality will be verified by the Kolmogorov-Smirnov test and the homogeneity of the variance will be validated by Levene’s test. Effect size will be calculated to estimate variance between moments through partial eta-squared. The cutoff values were defined as 0.02 for small effect size, 0.13 for moderate, and 0.26 for large effect sizes [36]. The chi-squared test will be used to check for a relationship between categorical variables. The effect size will be calculated using Cramer’s V test and interpreted based on the following cutoff values: 0.10 for a small effect, 0.30 for a medium effect, and 0.50 for a large effect. [36] No interim analyses will be conducted.

### Blinding

This study will involve the prescription of exercise sessions. To carry out a rigorous exercise prescription and to ensure adequate follow up of each patient, participants, the physical trainer (PA), the medical oncologist (AJ), and the nurse oncologist (ALA) will not be blinded to group assignment. Due to the lack of resources, only the evaluators acquiring the echocardiographic outcomes (resting LVEF and LV global longitudinal strain), circulating NT-proBNP levels, cardiorespiratory fitness, and recovery HR data, will be blinded to the group assignment.

### Limitations

There are some limitations to this study, which should be noted. First, we will only include patients followed at CHVNG/E. Second, due to the impossibility of blinding patients and some of the researchers to study group assignment, the open design of this study may influence the assessment retention rate of participants allocated to the control group. Third, we will stratify our sample based on age (under/over 50 years) and use of trastuzumab (yes/no). However, there are other risk factors associated with ARC, including total cumulative anthracycline dose, pre-existing cardiac disease, and treatment with mediastinal radiation, that should also be considered [19]. Although these hinderances should be acknowledged, we believe the findings from the present study will provide important data, which will be of relevance to the contemporary literature on this subject.

### Dissemination

The findings of this study will be reported in a doctoral thesis by the main author, will be submitted to a peer-reviewed journal for publication, and will be presented at relevant conferences and disseminated to the public in partnership with CHVNG/E, Universidade da Beira Interior (Covilhã, Portugal), and community stakeholders.

## Discussion

CVD is one of the leading causes of death among women with BC [3–5] and a foremost concern in the clinical practice of oncology [2]. According to Gernaat et al. [4], BC survivors have an absolute risk of dying from CVD that ranges from 1.6% to 10.4%. The recurrent use of cardiotoxic therapies and the presence of risk factors for the development and worsening of cardiovascular health problems are the main factors related to this phenomenon. The implementation of preventive strategies aiming to optimize cardiovascular care in BC survivors is therefore an emerging need. To date these include early identification of potential risk factors, the management of anthracycline cumulative dose (and possible use of ancillary therapies), or the use of drugs for heart failure (beta-blockers, angiotensin converting enzyme inhibitors, and angiotensin receptor antagonists) [21]. However, these approaches were mainly established to reduce the toxicity of therapeutic agents and ensure their efficacy, but not to provide a general preventive cardiovascular approach. It is thus pivotal to investigate the effects of non-pharmacological prevention strategies in order to counteract ARC and related complications in patients with BC.

Currently, exercise is recognized as a safe and effective, supportive intervention to improve physical function and HR-QOL in BC survivors during [19] or after treatment [20]. Furthermore, exercise has also been proposed as a potential tool to mitigate ARC in humans [21, 37], although this remains uncertain as it is mainly supported by data from animal studies and/or using less accurate biomarkers [22, 23]. In fact, there are limited data on whether the benefits of exercise also include protection from anthracycline-related cardiac damage in women with BC. To the best of our knowledge, Kirkham et al. [24] were the first to test this hypothesis by analyzing the efficacy of a single aerobic bout performed 24 h prior to each treatment. The authors observed positive effects on systemic outcomes (cardiac output, resting HR, body weight, and psychological symptoms), but did not observe relevant changes in echocardiographic outcomes (LV mass, LV ejection fraction, strain imaging, and the E/A ratio) or circulating cardiac biomarkers (troponin t and NT-proBNP). In a recent non-randomized trial, Howden et al. [26] verified that performing a 2-weekly supervised exercise program plus a weekly unsupervised aerobic session attenuated the reduction in peak Vo_2_ and the increase of troponin levels in women with BC undergoing AC-CT, when compared to a usual care group. However, these authors did not observe any relevant changes in the measured echocardiographic outcomes. So far, the overall clinical significance of exercise in preventing cardiac dysfunction associated with anthracycline treatment remains to be clarified in controlled clinical settings.

The present study will explore the cardioprotective role of exercise and potential mitigation of ARC, and establish the possible effect of exercise on different health outcomes in women with BC. A total of 90 adult women with early BC and prescribed AC-CT at the CHVNG/E will be enrolled. Recruitment will take place between 1 November 2018 and 31 November 2020 (expected date of recruitment completion). Follow up is expected to be completed by 30 June 2021.

We anticipate that the results of this study will add new knowledge to what the literature currently offers, clarifying the effects of a supervised exercise program on different established markers and more accurate biomarkers of cardiotoxicity in women with BC undergoing AC-CT. Furthermore, we expect that the findings from this study may help in future policies related to cancer care management and contribute to the ascertainment of the role of exercise programs during anti-neoplastic treatment in hospital.

## Trial status

Recruitment started on 1 November 2018 and is expected to be completed by 31 November 2020. Last edited, 11 January 2019.

## Additional file

Additional file 1: SPIRIT 2013 checklist: recommended items to address in a clinical trial protocol and related documents. (DOC 127 kb)

## Data Availability

The data that will support the findings of this study will be available on request from the corresponding author. The request will be analyzed by the research team and the ethics committee that ethically approved the study.

## Abbreviations

AC-CT: Anthracycline-containing chemotherapy
ARC: Anthracycline-related cardiotoxicity
AT: Anaerobic threshold
BC: Breast cancer
CHVNG/E: Centro Hospitalar de Vila Nova de Gaia/Espinho
CO2: Carbon dioxide
CRET: Cardiorespiratory Exercise Test
EORTC: European Organization for Research and Treatment in Cancer
FVC: Forced vital capacity
HF: High-frequency spectral component
HR: Heart rate
HR-QOL: Health-related to quality of life
HRV: Heart rate variability
IPAQ-SF: International Physical Activity Questionnaire-Short
LF: Low-frequency spectral component
LV: Left ventricular
LVEF: Left ventricular ejection fraction
MET: Metabolic equivalent
NT-proBNP: N-Terminal pro-brain natriuretic peptide
O2: Oxygen
peak V̇o_2_: Peak oxygen consumption
QOL-C30: Quality of Life C-30
RER: Respiratory exchange ratio
RMSSD: Root mean square of successive normal successive heartbeat variations
RPE: Rate of perceived exertion
R-R: Successive heartbeat variations
SDNN: Standard deviation of normal successive heartbeat variations
VE: Ventilatory volume
VVI: Velocity vector imaging
